# Biochemical and functional characterization of *Plasmodium falciparum* DNA polymerase δ

**DOI:** 10.1186/s12936-016-1166-0

**Published:** 2016-02-24

**Authors:** Jitlada Vasuvat, Atcha Montree, Sangduen Moonsom, Ubolsree Leartsakulpanich, Songsak Petmitr, Federico Focher, George E. Wright, Porntip Chavalitshewinkoon-Petmitr

**Affiliations:** Department of Protozoology, Faculty of Tropical Medicine, Mahidol University, 420/6 Rajvithi Road, Bangkok, 10400 Thailand; National Centre for Genetic Engineering and Biotechnology, National Science and Technology Development Agency, 113 Thailand Science Park, Pahonyothin Rd, Pathumthani, 12120 Thailand; Department of Molecular Tropical Medicine and Genetics, Faculty of Tropical Medicine, Mahidol University, Bangkok, 10400 Thailand; Institute of Molecular Genetics, CNR, 27100 Pavia, Italy; GLSynthesis Inc., One Innovation Drive, Worcester, MA 01605 USA

**Keywords:** *Plasmodium falciparum*, DNA polymerase δ, Drug target, Malaria, Biochemical characterization, Functional characterization

## Abstract

**Background:**

Emergence of drug-resistant *Plasmodium falciparum* has created an urgent need for new drug targets. DNA polymerase δ is an essential enzyme required for chromosomal DNA replication and repair, and therefore may be a potential target for anti-malarial drug development. However, little is known of the characteristics and function of this *P*. *falciparum* enzyme.

**Methods:**

The coding sequences of DNA polymerase δ catalytic subunit (PfPolδ-cat), DNA polymerase δ small subunit (PfPolδS) and proliferating cell nuclear antigen (PfPCNA) from chloroquine- and pyrimethamine-resistant *P. falciparum* strain K1 were amplified, cloned into an expression vector and expressed in *Escherichia coli*. The recombinant proteins were analysed by SDS-PAGE and identified by LC–MS/MS. PfPolδ-cat was biochemically characterized. The roles of PfPolδS and PfPCNA in PfPolδ-cat function were investigated. In addition, inhibitory effects of 11 compounds were tested on PfPolδ-cat activity and on in vitro parasite growth using SYBR Green I assay.

**Results:**

The purified recombinant protein PfPolδ-cat, PfPolδS and PfPCNA showed on SDS-PAGE the expected size of 143, 57 and 34 kDa, respectively. Predicted amino acid sequence of the PfPolδ-cat and PfPolδS had 59.2 and 24.7 % similarity respectively to that of the human counterpart. The PfPolδ-cat possessed both DNA polymerase and 3′–5′ exonuclease activities. It used both Mg^2+^ and Mn^2+^ as cofactors and was inhibited by high KCl salt (>200 mM). PfPolδS stimulated PfPolδ-cat activity threefolds and up to fourfolds when PfPCNA was included in the assay. Only two compounds were potent inhibitors of PfPolδ-cat, namely, butylphenyl-dGTP (BuPdGTP; IC_50_ of 38 µM) and 7-acetoxypentyl-(3, 4 dichlorobenzyl) guanine (7-acetoxypentyl-DCBG; IC_50_ of 55 µM). The latter compound showed higher inhibition on parasite growth (IC_50_ of 4.1 µM).

**Conclusions:**

Recombinant PfPolδ-cat, PfPolδS and PfPCNA were successfully expressed and purified. PfPolS and PfPCNA increased DNA polymerase activity of PfPolδ-cat. The high sensitivity of PfPolδ to BuPdGTP can be used to differentiate parasite enzyme from mammalian and human counterparts. Interestingly, 7-acetoxypentyl-DCBG showed inhibitory effects on both enzyme activity and parasite growth. Thus, 7-acetoxypentyl-DCBG is a potential candidate for future development of a new class of anti-malarial agents targeting parasite replicative DNA polymerase.

## Background

Malaria remains one of the major global public health problems in more than 100 endemic countries. Even though the numbers of malaria cases are decreasing, in 2013 there were still 198 million estimated cases globally and 584,000 deaths, mainly among sub-Saharan African children under 5 years of age [1]. *Plasmodium falciparum* is the most virulent human malaria parasite responsible for the majority of mortality cases. The emergence of anti-malarial resistance, in particular to artemisinins, has become a problem in malarial treatment and control [2–4]. Therefore, a better understanding of parasite metabolism, leading to identification of enzymes essential for its survival, should help in finding new targets for drug development.

One of the chemotherapeutic targets of interest is malarial DNA polymerase, which is an enzyme directly involved in polymerization of deoxynucleotides during replication and/or repair of cellular genetic material [5]. Eukaryotes possess 4 polymerases of the B-family, three of which, namely, DNA polymerase α (Pol α), DNA polymerase δ (Pol δ) and DNA polymerase ε (Pol ε), are essential enzymes for nuclear DNA replication [6]. Each enzyme plays a role in the replisome complex located at the replication fork, in which Pol δ replicates the lagging strand after it has been primed by Pol α [7]. Both Pol δ and Pol ε are distinguished from Pol α by their 3′–5′ proof-reading exonuclease activity, which allows removal of mis-incorporated deoxynucleotides, ensuring a high fidelity of DNA synthesis required for accurate genome replication [6].

Pol δ holoenzyme participates in replicative synthesis in concert with the processivity factor proliferating cell nuclear antigen (PCNA). Kinetic and binding studies have shown that PCNA increases Pol δ processivity as well as activity [8], possibly by forming a trimeric closed ring structure, which encircles the DNA and provides a sliding clamp for attachment of Pol δ [9]. In addition to its function in DNA replication, Pol δ plays a role in DNA repair and recombination [6]. In base excision repair (BER), one of DNA repair mechanisms of single-stranded DNA damage, Pol δ is involved in the long-path pathway, whereas Pol β plays a role in the short-path pathway [10]. Interestingly, the long-patch BER is predominate in *P. falciparum* while short-path BER is mainly found in humans [11].

Pol δ has been purified from a number of eukaryotes. In *Saccharomyces cerevisiae*, Pol δ is composed of three subunits: catalytic subunit Pol3p and structural subunits Pol31p and Pol32p [12–14]. In *Schizosaccharomyces pombe*, Pol δ consists of four subunits: Pol3, Cdc 1, Cdc27 and Cdm1 [15]. Human and mammalian enzymes initially were characterized as a heterodimer of p125 catalytic and p50 subunits [16, 17]. The p125 catalytic subunit is homologous to yeast Pol3 and Pol3p, whereas subunit p50 is a homologue of Cdc 1 and Pol31p [18, 19]. Later, two additional subunits of human and mammalian Pol δ were identified, namely, p68 and p12, displaying significant homology with *Schizosaccharomyces pombe* Cdc27 and Cdm1 respectively [20, 21]. Unlike mammalian Pol δ holoenzyme, formed by four subunits [21], only two subunits (p125 catalytic subunit and p50 small subunit) were identified in the Plasmodb sequence database.

Three types of *P. falciparum* DNA polymerases have been identified and characterized: nuclear Pol α and Pol β from parasite crude extract [22, 23] and Pol γ from parasite mitochondria [24]. *Plasmodium falciparum* (Pf)Pol δ gene of 3282 bp is located on chromosome 10 and encodes a protein of 1094 amino acids with 45 % similarity to *Saccharomyces cerevisiae* Pol δ [25, 26]. PfPol δ is expressed mainly in late trophozoite and schizont stages [27], but little is known about its enzymology and biochemical characteristics.

This study describes the cloning and expression of PfPol δ catalytic subunit (PfPolδ-cat) and the characterization of its activity in presence of its small subunit (PfPolδS) and proliferating cell nuclear antigen (PfPCNA). In addition, the in vitro inhibitory effects of 11 synthetic compounds on both recombinant PfPolδ-cat and parasite growth were evaluated for their potential as antiplasmodial drugs.

## Methods

### Parasites culture

*Plasmodium falciparum* strain K1, a chloroquine- and pyrimethamine-resistant strain from Thailand [28] was cultivated in RPMI 1640 medium (Invitrogen™, CA, USA) supplemented with 10 % human serum and human red blood cell (RBC) at 37 °C under an atmosphere of 5 % CO_2_. *Plasmodium falciparum* cultures containing mostly trophozoite and schizont stages were harvested when parasitaemia was >10 % by centrifugation at 500×*g* for 10 min at 25 °C.

### Construction of *PfPolδ*-*cat, PfPolδS* and *PfPCNA1* expression vectors

Genomic DNA of *P. falciparum* strain K1 was used as template to generate full-length *PfPolδ*-*cat*, *PfPolδS* and *PfPCNA1*. Amplification of *PfPolδ*-*cat* was carried out using *PfPolδ*-*cat*-forward (5′-CACCCATGGAAGAACTGAAAAC-3′) and *PfPolδ*-*cat*-reverse (5′-CCAATCCATTCTTAATGAGGT-3′) primers and Phusion^®^High-Fidelity DNA polymerase (Thermo Scientific, MA, USA) together with 30 cycles of PCR consisting of 98 °C for 1 min, 63 °C for 10 s and 72 °C for 105 s. *PfPolδS* was amplified using *PfPolδS*-forward (5′-CACCATGGACGAAAAAGTAACAAAC-3′) and *PfPolδ*-*p50*-reverse (5′-TTTGTCTTCGTCAATTTGAAAAGTC-3′) primers and Platinum^®^*Pfx* DNA polymerase (Invitrogen™) together with 35 cycles of PCR consisting of 95 °C for 1 min, 56 °C for 40 s and 72 °C for 2 min. *Pf*-*PCNA1* was amplified using primers previously described [29] and Phusion^®^High-Fidelity DNA polymerase together with 35 cycles of PCR consisting of 98 °C for 10 min, 58 °C for 5 s and 72 °C for 30 s. Amplicons were analysed either by 0.8 or 1.5 % agarose gel-electrophoresis. Amplified full-length *PfPolδ*-*cat* and *PfPolδS* was cloned into pBAD202/D TOPO^®^ and pET101/D TOPO^®^ expression vector (Invitrogen™), respectively. The constructed vectors, pBAD-PfPolδ-cat and pET-PfPolδS, were validated by nucleotide sequencing. Amplified full-length *Pf*-*PCNA1* was cloned into pQE-30 expression vector and named pQE-30-PfPCNA1.

### Expression and purification of PfPolδ-cat, PfPolδS and PfPCNA1

pBAD-PfPolδ-cat vector was transfected into *E. coli* LMG194 cells, which were grown in LB medium containing 50 μg/ml kanamycin at 37 °C with shaking until optical density of 600 nm reached 0.8. Then cells were induced by an addition of 0.002 % (w/v) L-arabinose and further incubated at 22 °C for 16–18 h. Cells were sedimented at 4 °C and then re-suspended in 3.5 volumes of cold lysis buffer (20 mM Tris–HCl pH 8.0 and 100 mM NaCl) per g of bacterial pellet. Cells were lysed using XL 2020 Sonicator^®^ Ultrasonic Processor XL (Heat System Inc., NY, USA), centrifuged at 10,000×*g* for 30 min at 4 °C. Supernatant was incubated with Q Sepharose Fast Flow (GE Healthcare, UK) on ice for 10 min to remove bacterial DNA and then applied onto 1-ml HisTrap HP column (GE Healthcare) prior equilibration with buffer A (20 mM Tris–HCl pH 8.0, 300 mM NaCl) containing 10 mM imidazole. The column was washed with buffer A containing 50 mM imidazole and enzyme was eluted with 250 mM imidazole-containing buffer A. Protein purity was analysed by SDS-PAGE.

pET-PfPolδS vector was used to transform *E. coli* BL21 (DE3) cells, which were grown in LB media containing 100 μg/ml ampicillin, and induced with 0.25 mM isopropyl β-D-1-thiogalactopyranoside (IPTG) at 22 °C for 16 h with shaking. Cells were collected and lysed as described previously. Supernatant was applied onto 1-ml HisTrap HP column and recombinant PfPolδS was eluted using a linear gradient of 20–250 mM imidazole in buffer A. Fractions of 0.25 ml were collected and analysed by SDS–PAGE.

pQE-30-PfPCNA1 was transfected into JM109 *E. coli* cells, which were grown in LB medium, induced with 1 mM IPTG at 25 °C for 16 h, collected and lysed as described above. Supernatant was incubated with Ni–NTA agarose affinity beads (QIAGEN, Hilden, Germany) at 4 °C for 2 h. The sample then was applied onto a gravity column, washed and protein eluted. The flow-through, wash and eluted fractions were collected, and analysed by SDS-PAGE. Protein concentrations were measured using Bradford assay [30] with bovine serum albumin (BSA) as standard.

### Western blotting and LC–MS/MS

After SDS-PAGE, proteins were electro-transferred onto Hybond-P PVDF membrane (GE Healthcare) and incubated at 4 °C overnight in phosphate-buffered saline (PBS) containing 5 % skim milk (blocking buffer). After washing three times with 0.05 % Tween-20 PBS buffer, membrane was incubated with mouse anti-His antibodies (Invitrogen) at 1:5000 dilution in blocking buffer at room temperature for 2 h. After washing, membrane was incubated with horseradish peroxidase-conjugated goat anti-mouse IgG for 1.5 h at room temperature and immunoreactive bands visualized using SuperSignal™ West Pico Chemiluminescent Substrate (Thermo Scientific). The expected protein bands were excised from gels and digested with trypsin. Patterns of peptide fragments and amino acid sequences were analysed using LC–MS/MS equipped with MASCOT software.

### DNA polymerase assay

DNA polymerase activity was assayed using activated calf thymus DNA (CT-DNA) (Sigma-Aldrich, MA, USA) as substrate. DNA polymerase assay was conducted in a 50-μl reaction mixture containing 10 μg of activated CT-DNA, 20 mM potassium phosphate buffer pH 8, 10 mM MgCl_2_, 2 mM DTT, 10 μg BSA, 50 μM each of dGTP, dATP and dCTP, 1 μM dTTP, 2.5 μM [α-^32^P]dTTP (800 Ci/mmol; PerkinElmer, MA, USA), and 42 nM PfPolδ-cat. After incubation for 1 h at 37 °C, the reactions were terminated by adding a 250-μl mixture of 20 mM EDTA, 0.1 mg/ml BSA and 100 μl of 50 % trichloroacetic acid (TCA), followed by sedimentation. The precipitate was then washed twice with 1 % TCA and [^32^P]dTMP incorporation was measured in a 1450 MicroBeta^®^ Trilux Liquid Scintillation Counter (Perkin Elmer). One unit of DNA polymerase activity is defined as the amount of enzyme that catalyzes the incorporation of 1 nmol of dTMP into DNA in 1 h at 37 °C.

### Effects of divalent ions and KCl on PfPolδ-cat activity

The effects of divalent cations, Mg^2+^ and Mn^2+^, on PfPolδ-cat activity were determined in the presence of 0.3 μM PfPolδ-cat and 0–50 mM MgCl_2_ or MnCl_2_ in the polymerase assay. The effect of KCl was determined over the range 0–400 mM.

### 3′–5′ exonuclease assay

The 3′–5′ exonuclease activity of PfPolδ-cat was measured from the release of [α-^32^P]dTMP from 3′ labelled poly(dA.dT) [16]. Substrate was prepared by incubating 125 μg/ml poly(dA.dT) with 5000 U/ml Klenow enzyme (New England Biolabs, MA, USA), 10 μM [α-^32^P]dTTP in 50 mM potassium phosphate pH 7.5, 5 mM MgCl_2_, 1 mM dAMP, and 0.5 mM β-mercaptoethanol. After incubation for 20 min at 37 °C, the reaction was termination by an addition of 10 mM EDTA and 1 M NaCl. The mixture was heated at 65 °C for 30 min and unincorporated [α-^32^P]dTTP removed employing AutoSeq™ G-50 dye terminator removal kit (GE Healthcare). For detection of exonuclease activity, a 30-μl reaction mixture containing 50 mM HEPES pH 7.0, 40 μg/ml BSA, 10 % glycerol, 2 mM MgCl_2_, 1.25 µg of 3′-labelled poly(dA.dT), and 0.2 μM PfPolδ-cat was incubated for 20 min at 37 °C and radioactivity measured as described above.

### Processivity assay

Processivity of PfPolδ-cat was determined using 500 ng of (dA)_1500_.(dT)_12_ (50:1 nucleotide ratio) as template-primer. The dT_12_ primer was at 5′ labelled with [γ-^32^P]dATP using T4 polynucleotide kinase and annealed to poly (dA)_1500_. Reaction mixture consisting of 20 mM Tris–HCl pH 9.0, 10 mM MgCl_2_, 0.2 mg/ml BSA, 2 mM DTT, 50 μM dTTP, and 42 nM PfPolδ-cat was incubated at 37 °C for 30 min. The product was precipitated with ethanol, dried, dissolved in sample buffer (95 % deionized formamide, 25 mM EDTA and 0.01 % bromophenol) and electrophoresed in 8 % polyacrylamide gel containing 7 M urea. Gel was exposed overnight to X-ray film at −80 °C.

### Effects of PfPolδS and PfPCNA1 on PfPolδ-cat activity

PfPolδS (0–1 μM) was added to a standard DNA polymerase assay containing 0.15 μg of polydA.oligodT and 1 μM PfPCNA1 and incubated at 37 °C for 30 min. The reaction mixtures were processed as described.

### Inhibitory effects of synthetic compounds on PfPolδ-cat activity

Inhibitory activity of 11 compounds consisting of substrate and nucleotide analogs and potential active site occupiers of PfPolδ-cat were compared with known Pol δ inhibitors, aphidicolin and N-ethylmaleimide (NEM). Stock solution (6 mM) of aphidicolin was prepared in dimethylsulfoxide (DMSO) and that of NEM (400 mM) in absolute ethanol. Stock solutions (10 mM) of *N*^*2*^-(4-butylphenyl)-2′-deoxyguanosine 5′-triphosphate (BuPdGTP), *N*^*2*^-(4-butylphenyl)- 2′-deoxyguanosine 5′-(*P*^*2*^*,P*^*3*^-carbonyltriphosphonate) (BuPdGMPPCOP), *N*^*2*^-ethyl-2′-deoxyguanosine 5′-triphosphonate (EtdGTP), *N*^*2*^-hexyl-2′-deoxyguanosine 5′-triphosphate (HexdGTP) and Acyclovir triphosphate (ACV-TP) were prepared in sterile distilled water, while those of 2-amino-4-chloro-6-(3,4-dichloroanilino)pyrimidine, 2-amino-4-chloro-6-(3,5-dichloroanilino)pyrimidine, N^2^-(3,4-dichlorobenzyl)guanine (DCBG), N^2^-(3-fluoro,4-chlorobenzyl)guanine, 3-(4-hydroxybutyl)-6-(3-ethyl-4-methylanilino)uracil (HB-EMAU), and 7-acetoxypentyl-(3,4-dichlorobenzyl)guanine (7-acetoxypentyl-DCBG) were prepared in DMSO. All stock solutions were stored at −20 °C until used. Test concentrations of compounds were prepared by diluting stock solution with 10 mM Tris–HCl pH 8.0 and evaluated in triplicate. Compounds were added directly to the reaction mixtures except for NEM that was pre-incubated with enzyme for 30 min on ice before addition to the reaction mixture. Polymerase activity assays were conducted as described above.

### Inhibition of intra-erythrocytic *P. falciparum* growth in culture

*Plasmodium falciparum* K1 strain was synchronized at ring stage using 5 % D-sorbitol treatment and then mixed with culture medium containing RPMI 1640 medium supplement with 10 % human serum. Twofold serial dilutions of each test compound were evaluated in triplicate. Parasite growth was determined by a SYBR Green I-based assay [31, 32]. Dose–response curves and IC_50_ values were obtained using SigmaPlot 12.0.

## Results

### Expression and purification of recombinant PfPolδ-cat, PfPolδS and PfPCNA1

The 3285-bp full-length *PfPolδ*-*cat* of *P*. *falciparum* K1 strain was successfully amplified and cloned employing pBAD202/D TOPO^®^ expression vector. Its nucleotide sequence showed 99 % identity to that of *P. falciparum* 3D7 (NCBI reference sequence XM_001347414.1). The deduced amino acid sequence (1094 amino acids) of PfPolδ-cat is 84 and 59.2 % similar to that of *Plasmodium vivax* and humans, respectively (Table 1).

**Table 1:** Amino acid sequence similarity of PfPolδ-cat compared with Polδ from other organisms

Organism	Accession number of NCBI protein reference sequence	Similarity (%)
*P. falciparum 3D7*	XP_001347450.1	100
*Plasmodium vivax Sal*-*1*	XP_001612703.1	84.0
*Homo sapiens*	NP_001243778.1	59.2
*Schizosaccharomyces pombe*	NP_596124.1	62.4
*S. cerevisiae S288c*	NP_010181.2	59.4
*Toxoplasma gondii*	XP_002365027.1	57.0
*Mus musculus*	NP_035261.3	60.4

**Fig. 1:**
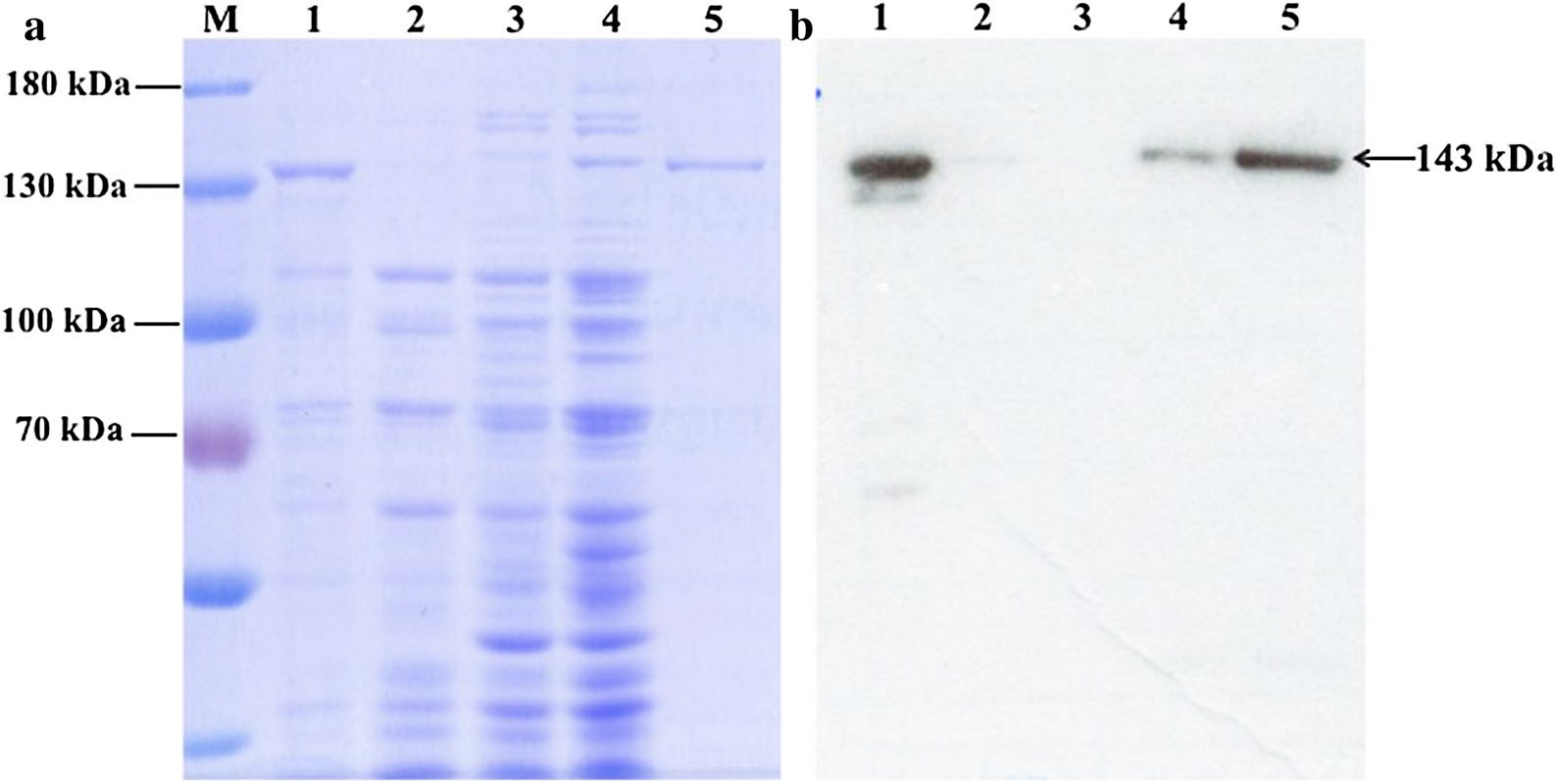
SDS–PAGE and western blot analysis of PfPolδ-cat expression. **a** SDS-PAGE (7.5 %) and **b** western blot analysis of PfPolδ-cat. Anti-His antibody was used for western blot analysis. *Lane M* molecular weight markers; *lane 1* induced whole cell of *E. coli* LMG194 carrying pBAD vector with lacZ gene containing His_6_ sequence; *lane 2* non-induced whole cell of *E. coli* LMG194 carrying pBAD vector; *lane 3* non-induced whole cell of *E. coli* LMG194 carrying *PfPolδ*-*cat* vector; *lane 4* induced whole cell of *E. coli* LMG194 carrying *PfPolδ*-*cat* vector; *lane 5* HisTrap HP column purified protein

Recombinant PfPolδ-cat was expressed as thioredoxin-PfPolδ-cat-His_6_ fusion protein in *Escherichia coli* LMG194 and purified by Ni^2+^ affinity chromatography, having the expected size of 143 kDa. The method yielded 0.48 mg of protein/l culture. Western blot analysis using His-specific antibody indicated a single PfPolδ-cat band of the expected 143 kDa (Fig. 1). After excision from gel, trypsin digestion and amino acid sequence analysis using LC–MS/MS, seven peptides were obtained that matched the sequence of *P. falciparum* 3D7 Polδ-cat with ion scores of 100, indicating identity or extensive homology (*p* value < 0.05).

**Fig. 2:**
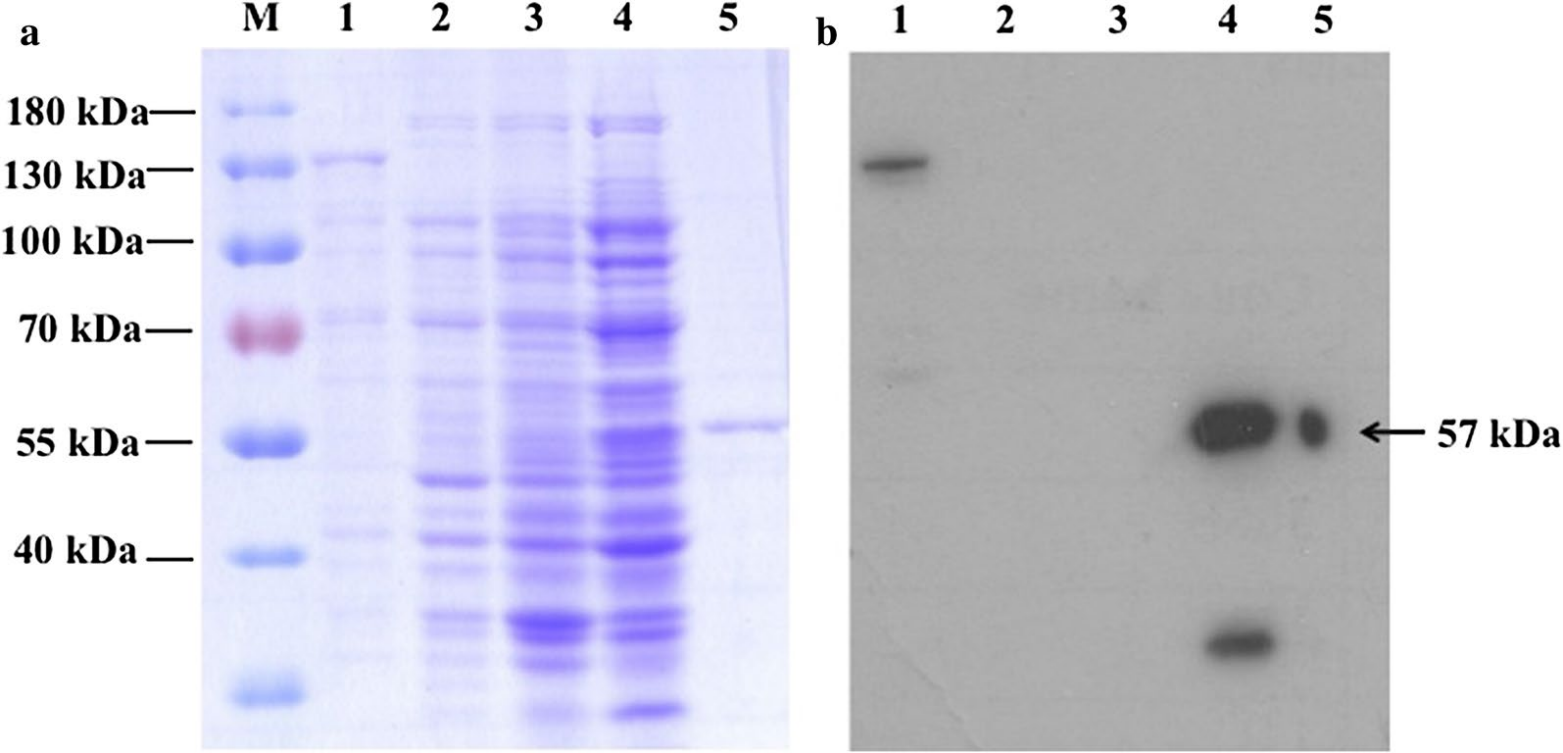
SDS–PAGE and western blot analysis of PfPolδS expression. **a** SDS–PAGE (10 %) and **b** western blot analysis of PfPolδS. Anti-His antibody was used for western blot analysis. *Lane M* molecular weight markers; *lane 1* positive control vector with lacZ gene containing His_6_ sequence;* lane 2* non-induced whole cell of *E. coli* BL21 carrying pET vector; *lane 3* non-induced whole cell of *E. coli* BL21 carrying PfPolδS vector; *lane 4* induced whole cell of *E. coli* BL21 carrying PfPolδS vector; *lane 5* HisTrap HP column purified protein

**Fig. 3:**
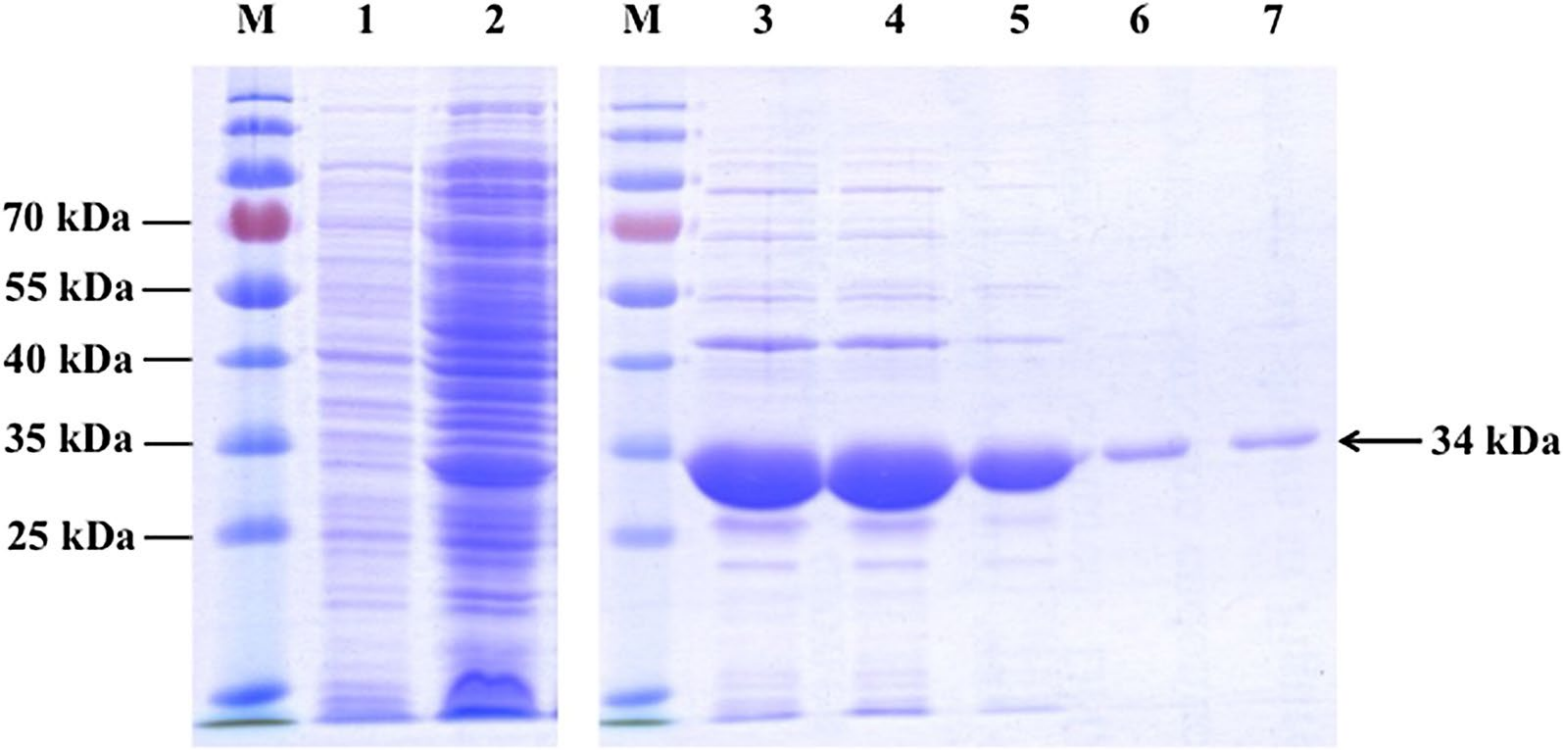
Analysis of recombinant PfPCNA1 expression by 12 % SDS–PAGE. Gel was stained with Coomassie blue R250. *Lane* *M* molecular weight markers; *lane 1* non-induced whole cells of *E. coli* JM109 expression host; *lane 2* total soluble protein from 1 mM IPTG-induced *E. coli* JM109 carrying PfPCNA1-constructed vector; *lanes 3–7* elution fractions from Ni–NTA agarose purified protein

In addition to PfPolδ-cat, PfPolδS and PfPCNA1 were expressed and purified. PfPolδS was expressed under the regulation of T7 promoter and carried His_6_ at C-terminus. Affinity purified PfPolδS with a molecular mass of 57 kDa was obtained (Fig. 2), and its identity was confirmed by LC–MS/MS, which yielded five peptides matching *P. falciparum* 3D7 DNA polymerase δ small subunit with ion scores of 168. The deduce amino acid sequence of PfPolδS reveals 49.1 and 24.7 % similarity with that of *P. vivax* and human PolδS, respectively (Table 2). As PfPCNA1 has been characterized previously [27], the reported protocols were adapted to obtain a purified protein of 34 kDa with final yield of 4.35 mg/l culture (Fig. 3).

**Table 2:** Amino acid sequence similarity of PfPolδS compared with other organisms

No.	Organism	GenBank accession no.	Similarity (%)
1	*P. falciparum* 3D7	CAB11105.1	85.9
2	*P. vivax*	EDL43374.1	49.1
3	*P. knowlesi* strain H	CAQ39738.1	51.4
4	*H. sapiens*	AAC50216.1	24.7
5	*S. pombe*	CAB11679.1	19.4
6	*Entamoeba dispar*	EDR22929.1	16.9
7	*Candida dubliniensis* CD36	CAX41815.1	18.7
8	*Culex quinquefasciatus*	EDS33455.1	24.0

### Biochemical characterization of PfPolδ-cat

**Fig. 4:**
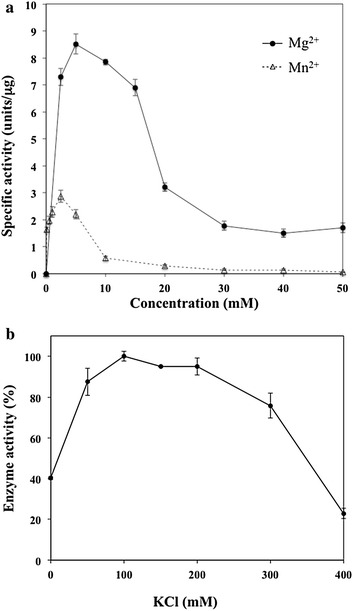
Effects of divalent cation cofactors and KCl concentrations on PfPolδ-cat. DNA polymerase activity of PfPolδ-cat was determined using CT-DNA as template-primer. **a** PfPolδ-cat activities were investigated in the presence of MgCl_2_ (*circle*) and MnCl_2_ (*triangle*) and presented as specific activity. **b** concentrations of KCl were varied as indicated. Maximal activity of enzyme is designated as 100 %. Data are shown as mean ± SD

Recombinant PfPolδ-cat exhibited both DNA polymerase (Fig. 4) and 3′–5′ exonuclease activity, the latter property being based on the release of [α-^32^P]dTMP from 3′-labelled-poly(dA.dT), reducing TCA insoluble material by 96.6 ± 1.2 % after 20 min incubation at 37 °C. PfPolδ-cat polymerase activity required presence of divalent metal ions, Mn^2+^ or Mg^2+^, with maximal polymerase activity being achieved at 2.5 and 5 mM respectively (Fig. 4a). Although both divalent cations activated polymerase activity, PfPolδ-cat was approximately threefold more active in the presence of Mg^2+^ than Mn^2+^ at their respective optimal concentration. As regards KCl requirement, PfPolδ-cat showed maximal polymerase activity at 100 mM KCl and activity decreased when KCl was >200 mM (Fig. 4b).

### Effects of PfPolδS and PfPCNA1 on PfPolδ-cat polymerase activity

**Fig. 5:**
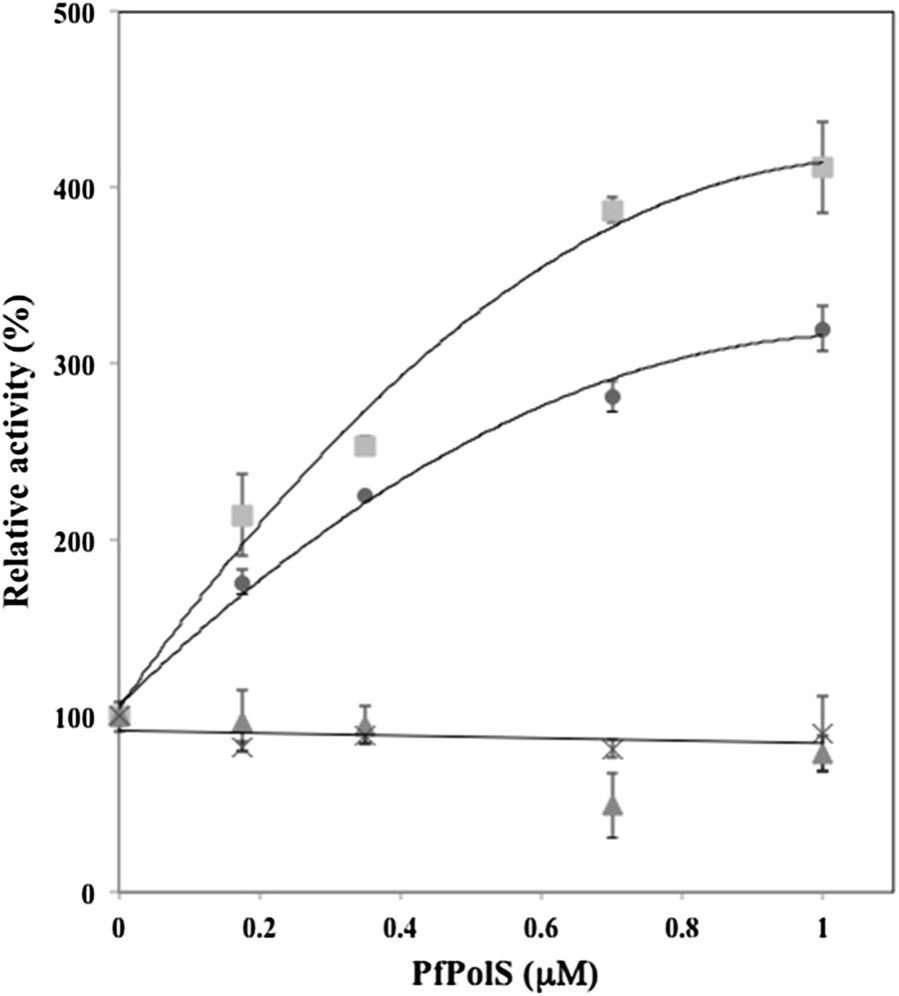
Stimulation of PfPolδ-cat DNA polymerase activity. Assays were performed using poly(dA).oligo(dT) as template-primer. The effects of PfPolδS (*circle*), PfPolδS in presence of 1 μM PCNA (*square*) were compared with 0.5 mg BSA (*triangle*) and heat-treated PfPolδS (*cross*) as negative controls. Data are shown as mean ± SD

**Fig. 6:**
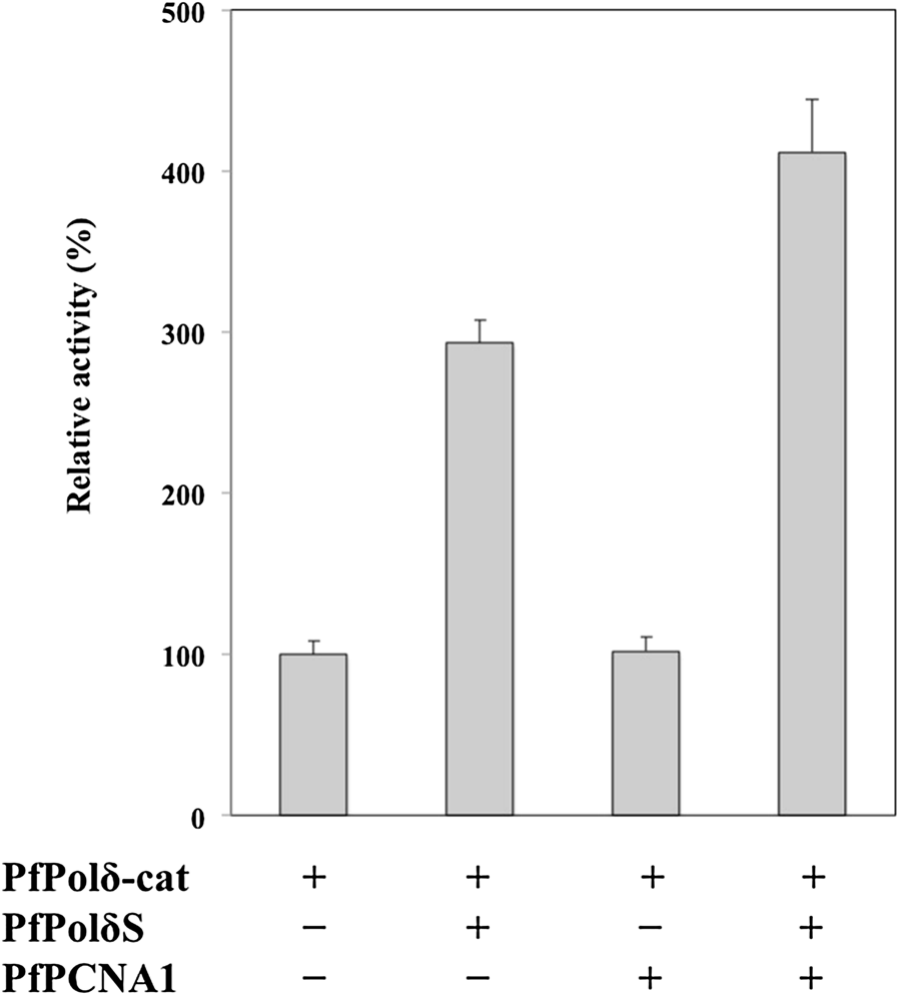
Effects of recombinant PfPolδS and PfPCNA1 on PfPolδ-cat activity. DNA polymerase activity was determined using poly(dA).oligo(dT) as template-primer. Activity of PfPolδ-cat alone is presented as 100 % and data are shown as mean ± SD

The effect of PfPolδS on PfPolδ-cat polymerase activity was determined by adding recombinant PfPolδS to a standard polymerase assay in the presence or absence of PfPCNA1. PfPolδS was able to stimulate PfPolδ-cat DNA polymerase activity threefold, which was abrogated in the presence of heat-treated PfPolδS (Fig. 5). However, the presence of PfPCNA1 alone did not alter PfPolδ-cat polymerase activity, but there was a fourfold increase in activity was when both PfPolδS and PfPCNA1 were present (Fig. 6).

### Processivity of PfPolδ-cat

**Fig. 7:**
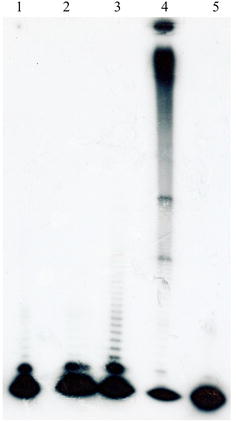
Effects of PfPolδS and PfPCNA1 on processivity of PfPolδ-cat. *Lane 1* products of PfPolδ-cat; *lane 2* products of PfPolδ-cat in the presence of PfPolδS; *lane 3* PfPolδ-cat in the presence of PfPolδS and PfPCNA1; *lane 4* products of *E.coli* Klenow enzyme as positive control; *lane 5* negative control

PfPolδ-cat processivity was investigated using (dA)_1500_.(dT)_12_ as substrate in comparison with that of *E. coli* DNA polymerase Klenow fragment. In the presence of PfPolδS and PfPCNA1, PfPolδ-cat produced longer products (higher processivity) than in their absence (Fig. 7).

### Effects of inhibitors on DNA polymerase activity of PfPolδ-cat and parasite growth

Of the 11 synthetic compounds tested on PfPolδ polymerase activity only four showed inhibitory effects, namely, BuPdGTP (IC_50_ = 38 µM), 7-acetoxypentyl-DCBG (IC_50_ = 55 µM), 2-amino-4-chloro-6-(3′,4′-dichloroanilino)pyrimidine (IC_50_ = 104 µM) and 2-amino-4-chloro-6-(3′, 5′-dichloroanilino)pyrimidine (IC_50_ = 185 µM), but still less potent than aphidicolin (IC_50_ = 11.8 μM) and NEM (IC_50_ = 8.6 mM), as seen with other eukaryotic Pol δ [14–16] (Table 3). These four compounds also inhibited *P*. *falciparum* K1 strain growth in culture, with IC_50_ values ranging from 3.8 to 85.6 µM (Table 3). However, DCBG, N^2^-(3-fluoro,4-chlorobenzyl)guanine and HB-EMAU exhibited inhibitory effects on *P. falciparum* growth, with IC_50_ values of 8.8, 7.4 and 10.2 μM, respectively.

**Table 3:** Inhibitory effects (IC_50_) of compounds on PfPolδ-cat activity and in vitro malaria parasite growth

Compound	IC_50_ (µM)	
	PfPolδ-cat	*P. falciparum*
BuPdGTP	38.0 ± 1.7	85.6 ± 3.7
7-acetoxypentyl-DCBG	55.0 ± 3.4	4.1 ± 0.2
2-amino-4-chloro-6-(3′,4′-dichloroanilino)pyrimidine	104.0 ± 5.6	3.8 ± 0.3
2-amino-4-chloro-6-(3′, 5′-dichloroanilino)pyrimidine	185.0 ± 7.7	34.4 ± 0.4
BuPdGMPPCOP	Inactive^a^	173.4 ± 2.7
EtdGTP	Inactive^a^	157.8 ± 3.4
HexdGTP	Inactive^a^	86.4 ± 0.2
Acyclovir triphosphate	Inactive^a^	347.2 ± 8.3
DCBG	Inactive^a^	8.8 ± 0.5
N^2^-(3-fluoro,4-chlorobenzyl)guanine	Inactive^a^	7.4 ± 0.3
HB-EMAU	Inactive^a^	10.2 ± 0.4

^a^<20 % inhibition at 100 μM

## Discussion

Since 1976, when, for the first time, Pol δ was described in bone marrow as a novel DNA polymerase possessing a 3′–5′ proofreading exonuclease activity [33], eukaryotic Polδs have been purified and characterized from several organisms [16, 17, 34], with the exception of malarial parasites.

PfPolδ partially purified from parasite crude extract using Hitrap Capto Q and Hitrap Heparin columns in a fast protein liquid chromatography (FPLC) system exhibited 3′–5′ exonuclease activity and was sensitive to aphidicolin and NEM (unpublished). However, possible co-purification of PfPolε could not be rule out. Subsequent purification of PfPolδ to near homogeneity was hampered by very low recovery yield and a lack of Pol δ-specific affinity column able to separate it from PfPolε. Therefore, in this study a DNA recombinant approach was used to study PfPolδ catalytic subunit, in the presence of its small subunit PfPolδS and PfPCNA. Moreover, recombinant techniques provided sufficient amounts of enzyme to allow testing as a potential anti-malarial drug target.

Recombinant PfPolδ was successfully cloned and heterologously expressed in *E. coli*, but the expressed protein was produced in an insoluble form at 37 °C. The expression condition was optimized by reducing expression temperature, which usually increases soluble protein yield [35]. The size of expressed PfPolδ-cat was 126 kDa, comparable to 130 kDa of *E. coli* Pol III [36] and 125 kDa of purified human enzyme [16]. Tandem mass spectrometry of trypsinized recombinant PfPolδ revealed seven peptides that showed high homology with the sequence of DNA polymerase δ catalytic subunit of *P. falciparum* strain 3D7. Characterization of recombinant PfPolδ-cat showed that it possesses both DNA polymerase activity and 3′–5′ exonuclease activity, as found in other mammalian Polδs [16, 17, 34].

PCNA functions as a processivity factor for Pol δ by forming a molecular sliding clamp and also plays a crucial role in DNA transactions where it acts as a scaffold for the recruitment and organization of protein complexes involved in both DNA replication and repair [19]. A previous study of protein–protein interactions of human Pol δ-PCNA complex suggested that the interaction between Pol δ and PCNA likely happens through multiple contacts via its four subunits, p12, p50, p68, and p125 [19, 37]. In this study, PfPolδ-cat polymerase activity was stimulated threefolds with the addition of PfPolδS and fourfolds in the presence of both PfPolδS and PfPCNA. The magnitude of malarial enzyme activity stimulation is comparable to those obtained from examination of the effect of human recombinant p50 on the activity of DNA polymerase δ, showing that p50 is able to slightly stimulate (about twofold) the activity of the recombinant 125 kDa catalytic subunit in the absence of PCNA, while in the presence of PCNA polymerase activity is stimulated about fivefold [38]. In addition, a combination of PfPolδ-cat, PfPolδS and PfPCNA1 demonstrated highest processivity compared with individual protein or incomplete combination. These findings are consistent with previous report indicating that small subunit p50 is required for mediation of the interaction of human Pol δ catalytic subunit (p125) with PCNA [19]. In this study, replication factor C (RFC) or clamp loader of *P. falciparum* was not used in the assay with PfPolS and PCNA, and thus higher activity of PfPolδ-cat would be expected upon addition of RFC as the latter is responsible for loading PCNA onto DNA during DNA replication [6]. In addition to its important function in DNA replication, a role of PfPolδ-cat in base excision repair should be investigated when additional enzymes or proteins in parasite BER pathway become available.

All DNA polymerases use the same two metal cations (usually Mg^2+^) as co-factors for dNTP polymerization. In this study, PfPolδ-cat was able to use both Mg^2+^ and Mn^2+^ and could be activated by 5 mM Mg^2+^ as found for both human and calf thymus Pol δ [16, 17]. However, in the case of Mn^2+^, optimal concentration (2.5 mM) required by the parasite enzyme was five- to eightfold higher than that optimal for human and calf thymus Pol δ (0.3–0.5 mM) [39].

PfPolδ activity was differently affected by salt concentrations compared with calf thymus and human recombinant enzymes. The maximal polymerase activity of PfPolδ-cat was at 100 mM KCl and declined at higher concentrations (>200 mM). These findings are different from those observed with recombinant human and calf thymus Pol δ, where only 50 and 38 % of enzyme activity respectively was found at 50 mM KCl [16, 39]. Unlike the human and calf thymus enzymes, Pol δ of Drosophila is slightly stimulated by low KCl concentration (25 mM) [40]. Recombinant Pol3 of *Schizosaccharomyces pombe* shows maximal activity at 240 mM KCl, whereas its native form is sensitive to high salt concentration [41]. It is possible that KCl may help stabilize protein at a concentration suitable for function or to reduce its self-aggregation.

Only 4 of 11 synthetic compounds showed inhibitory effects on PfPolδ-cat activity when compared with known DNA polymerase inhibitors such as aphidicolin and NEM. The most potent inhibitor of PfPolδ-cat was BuPdGTP, which strongly inhibited mammalian Pol α compared with Pol δ and ε [42]. In contrast to BuPdGTP, 2-amino-4-chloro-6-(3′,4′-dichloroanilino)pyrimidine showed low inhibitory effect on PfPolδ-cat but was the most potent inhibitor of parasite growth in culture. Inhibition of PfPolδ-cat activity by these two compounds did not directly correlate with parasite growth inhibition, suggesting that they may have different cell permeability and metabolic properties. However, 7-acetoxypentyl-DCBG was the most potent inhibitor of both PfPolδ-cat activity and parasite growth. Recently, 7-acetoxypentyl-DCBG was shown to be a potent antibiotic, showing an MIC of 1.25 μg/ml and a clear dose–response effect (80 % mice survived after treatment with an IP dose of 60 mg/kg) [43]. Taken together, 7-acetoxypentyl-DCBG is a promising starting template for future rational design of a selective inhibitor against PfPolδ and may lead to development of novel anti-malarial agents.

## Conclusions

Recombinant PfPolδ-cat, PfPolδS and PfPCNA1 were successfully expressed heterologously. PfPolδ-cat contains both DNA polymerase and 3′–5′ exonuclease activity as found in the human counterpart. However, recombinant PfPolδ-cat and PfPolδS differ from human enzymes in their deduced amino acid sequences. A combination of PfPolS and PfPCNA clearly stimulated PfPolδ-cat DNA polymerase activity and processivity. Recombinant PfPolδ-cat was inhibited by two guanine analogs, namely, BuPdGTP and 7-acetoxypentyl-DCBG. Furthermore, 7-acetoxypentyl-DCBG was demonstrated to be a potent inhibitor of in vitro malaria parasite growth. Analogs of this compound should further be developed into more potent anti-malarial drugs.
